# Herpetic esophagitis in immunocompentent host: cases report

**DOI:** 10.1186/s12879-020-05328-5

**Published:** 2020-08-17

**Authors:** Alba M. Diezma-Martín, Esther Gigante-Miravalles, Juan Diego Castro Limo, Carlos Andrés Quimbayo Arcila, Juan José Puche Paniagua

**Affiliations:** 1 Department of Neurology, Complejo Hospitalario de Toledo, Toledo, Spain; 2 Department of Cardiology, Complejo Hospitalario de Toledo, Toledo, Spain; 3 Department of Gastroenterology, Complejo Hospitalario de Toledo, Toledo, Spain; 4 Department of Pathology, Complejo Hospitalario de Toledo, Toledo, Spain; 5 Department of Medicine, Complejo Hospitalario de Toledo, Toledo, Spain

**Keywords:** Esophagitis, Herpes simplex, Acyclovir

## Abstract

**Background:**

Herpetic esophagitis (EH) usually affects those who are immunocompromised and is uncommon in immunocompetent patients. In these cases, EH may occasionally present as an acute and self-limited illness. Such cases are rare and only a few have beenreported and limited published reviews exist making the benefits of antiviral therapy in immunocompetent patients unknown.

**Case presentation:**

We report four cases of young patients who presented dysphagia, odynophagia and epigastric pain. Endoscopic findings revealed lesions in the distal esophagus and histopathological changes compatible with herpes virus infection confirmed by viral DNA in every case. After treatment, every patient showed significant improvement and tolerated oral intake after discharge.

**Conclusions:**

In this publication, we present four immunocompetent patients with EH, without relevant alterations in laboratory workup and with negative HIV status. This disease is infrequent in patients with such characteristics and there are few cases published. In order to better understand this pathology, we present the symptoms, the endoscopic alterations and the clinical evolution with treatment. In our series, 50% of patients had serology compatible with acute HVS type 1 infection, 25% had a subacute infection pattern (IgM and IgG positive antibodies) and in another 25% of patients, serology was not done. No patient presented leukocyte alterations, while all patients presented with anatomopathological findings compatible with acute herpetic esophagitis and responded to acyclovir therapy.

## Background

Herpetic esophagitis (EH) caused by herpes simplex virus (HSV) usually affects immunocompromised patients, as a primary infection or as a reactivation of a previous infection. It is rarely present in immunocompetent hosts [1]. In these patients, the infection is usually related to a primo-infection and is a self-limited condition [2]. EH due to HSV type 1, is more common than type 2 [3]. Some comorbidities and predisposing factors are described in many studies in relation with this entity in immunocompetent population, such as alcohol consumption, eosinophilic esophagitis, malnutrition or frequent use of corticosteroids [4]. These findings suggest that the disease process is more likely to occur in the presence of esophageal pathology (such as severe gastroesophageal reflux disorder biopsy sites) as the virus is more likely to infect traumatized tissue; although there are many cases where there is no identified damage to the mucosal integrity [5]. Clinically, it usually manifests as odynophagia and or dysphagia. In addition, fever and retrosternal pain may be present. These clinical manifestations can coexist with herpes lesions on the lips and or ulcers at the oropharyngeal level. Complications (such as bleeding and esophageal perforation) and recurrences in immunocompetent patients are much less frequent than in immunosuppressed patients [6]. This pathology predominates in women and young patients under the age of 40 [4]. A high degree of suspicion and early upper gastrointestinal endoscopy are required for diagnosis. In the endoscopy, we can find vesicles in different evolutionary stages of the disease. At the beginning we may see ulcers surrounded by healthy mucosa, then big “volcano” ulcers with exudates [7]. Lesions are usually resolved spontaneously, but acyclovir can accelerate their resolution and improve symptoms [2, 8, 9].

Our objective in this case report was to review the clinical, endoscopic, serological and anatomopathological characteristics of this disease, along with its evolution and results with symptomatic treatment in immunocompetent patients. We present our experience with four young patients with EH from our hospital.

## Cases presentation

### Case 1

A 22 year-old woman to emergency department presented with severe retrosternal pain, odynophagia, dysphagia, nausea, and fever reaching up to 39,5 °C. The patient had prior contact with VZV lesions in a relative affected with this infection previously. Her past medical history was not significant. Laboratory testing showed high level of IgM and IgG titers for HVS type 1 and 2. Esophagogastroduodenoscopy revealed small white ulcers on normal mucosa that covered the entire esophageal surface with confluent exudate in the distal esophagus. The histology of the biopsy specimen showed multiple cells with intranuclear inclusions consistent with herpetic infection. PCR was positive for HSV-1. The patient was treated with oral acyclovir for eight days with a favorable clinical evolution.

### Case 2

A 21 year-old man was admitted to our hospital complaining of odynophagia, chest pain, fever, and oropharyngeal lesions. His past medical history was not noteworthy. Laboratory testing was significant only for high levels of IgM titers for HVS type 1. His HIV status was negative. Esophagoscopy revealed multiple linear ulcers in the upper third of the esophagus. Microscopic examination of the esophageal biopsy showed both acute and chronic inflammation. PCR was positive for HSV-1. In response to these results, the patient was treated with oral acyclovir for eight days. The symptoms resolved with this therapy in few days.

### Case 3

A 15 year-old man who had presented with a fever of 39 °C, epigastric pain, odynophagia, chest pain and vomiting. In jugal mucosa whitish lesions were present compatible with candida and a small cervical adenopathy was observed. His medical history indicated autism spectrum disorder and eating disorder. Serology was positive for IgM and IgG antibodies by HSV-1 and 2 and negative for HIV and candida. Gastroscopy showed longitudinal, no confluence ulcerations with a fibrin bottom in over half of the distal esophagus, which were later biopsied. Finally, histopathology presented esophageal epithelium with ulcerations, antral gastric mucosa with moderated superficial chronic inflammation without intestinal metaplasia and *H. pylori* bacils. PCR was positive for HSV-1. The patient received symptomatic treatment without antiviral treatment. The eradication treatment against *H. pylori* was prescribed after resolution of the herpetic infection.

### Case 4

A 23 year-old man who had presented with epigastric pain associated to pyrosis, acid regurgitation, fever and dysphagia to solid food. His medical history indicated cereal allergy and exercise anaphylaxis. The gastroscopy showed longitudinal and superficial ulcerations with geographical borders from gastroesophageal union to cervical esophagus. Histopathology showed erosions and superficial ulcerations in esophageal epithelium. PCR was positive for HSV-1 DNA and negative for HIV. He received treatment with proton pump inhibitors and acyclovir for ten days with clinical improvement. The following month, a control gastroscopy was done, showing lineal and erythematous scars which ascended to the medium esophagus. Biopsies were negative for HSV-1 DNA.

## Discussion and conclusion

EH is a pathology which usually occurs in immunocompromised patient, being extremely uncommon in immunocompetent patients. In this publication, we present four immunocompetent patients with EH, without relevant alterations in laboratory workup and with negative HIV status. Clinical characteristics of EH in immunocompetent host are odynophagia, dysphagia, heartburn, epigastric pain or chest pain [1, 8]. All patients presented some of these symptoms. As prodromes, fever, nausea, vomiting or cough had been described in the literature [3]. Our findings show 100% of patients presented with fever and none presented with a cough or nausea. Oropharyngeal lesions were less frequent [1, 8]; however, two patients in the series exhibited these types of lesions. In some cases, due to the appearance of the lesions, candida esophagitis may be suspected, more frequently in immunocompromised cases or with eosinophilic esophagitis background, which is a frequent mistake. Only in one of our patients did this confusion initially occur, but the lesions where ultimately classified as herpetic disease.

In previous studies, it has been questioned whether EH might be the trigger to develop eosinophilic esophagitis (EoE) in patients who were already genetically predisposed for this condition, presenting in many cases a history of atopy or allergies [6, 9]. Our findings did not show this association. It is believed that the breakdown of the esophageal mucosa in relation with HVS and the activation of the immune system can serve as a trigger for the development of EoE [10] [11].

There are other comorbidities and predisposing factors described, such as close exposure to HSV lesions in a relative before the onset of the symptoms. It has been seen to be responsible for up to 21.6% cases depending on the series [1]. Only one of our patients with EH, without relevant alterations in laboratory workup and with negative HIV status, had previous contact with VVZ lesions in an infected relative, which was not described as a risk factor in previous studies.

As for the analytical alterations, the absence of leukocytosis in immunocompetent patients was frequent although it was possible to see atypical active lymphocytes in the blood smear [3]. In our series, a blood smear was not carried out in any patient because none of them presented leukocyte alterations. On the other hand, serology usually showed an acute infection pattern (positive IgM antibody, negative IgG antibody) for HSV type 1 infection more frequently or HSV type 2 infection with possible seroconversion up to 3 to 4 weeks later [1]. In our series, 50% of patients had serology compatible with acute infection, 25% had a subacute infection pattern (IgM and IgG positive antibodies) and another 25% did not undergo serology testing. In all cases serology for HIV was negative. In one case, serology was positive for VZV. This findings may be due to ELISA testing being that in occasions it may react with antibodies to different virus types of same family as is the case for HSV-1 and 2, VZV or CMV [9, 12].

**Fig. 1:**
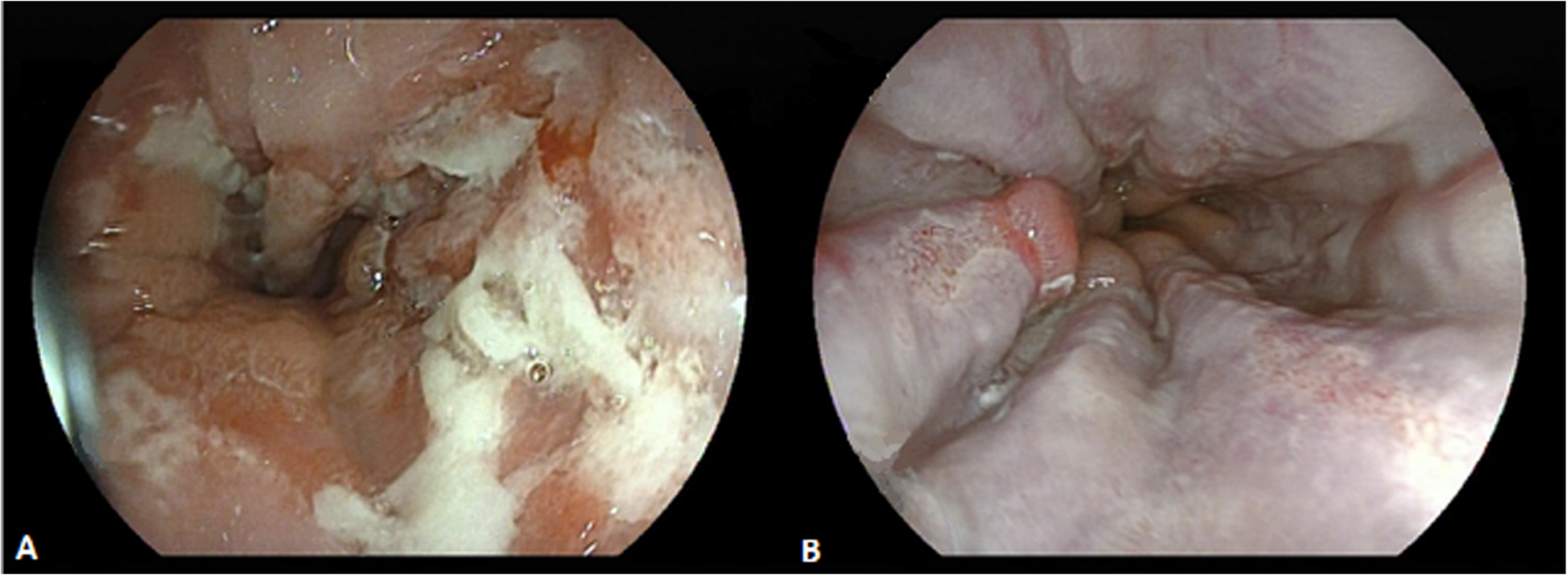
A) Endoscopy image from case 1 showing multiple ulcerations with exudate throughout the distal esophagus. B) Endoscopy image from case 2 where superficial ulcerations of geographical edges that ascend longitudinally from the distal esophagus are observed

EH commonly affects the middle and distal esophagus (68.3%) and less frequently the proximal esophagus [5, 6] and interesting to note that 75% of patients presented with proximal esophageal lesions [6]. Depending on the moment of endoscopy, the lesions were initially vesicles which evolved posteriorly to multiple superficial circumscribed ulcers with a volcanic appearance, which ascend from distal esophagus, in a friable mucosa (Fig. 1). As in case 1, exudes were also presented on multiple occasions (Fig. 1) [1, 6].

**Fig. 2:**
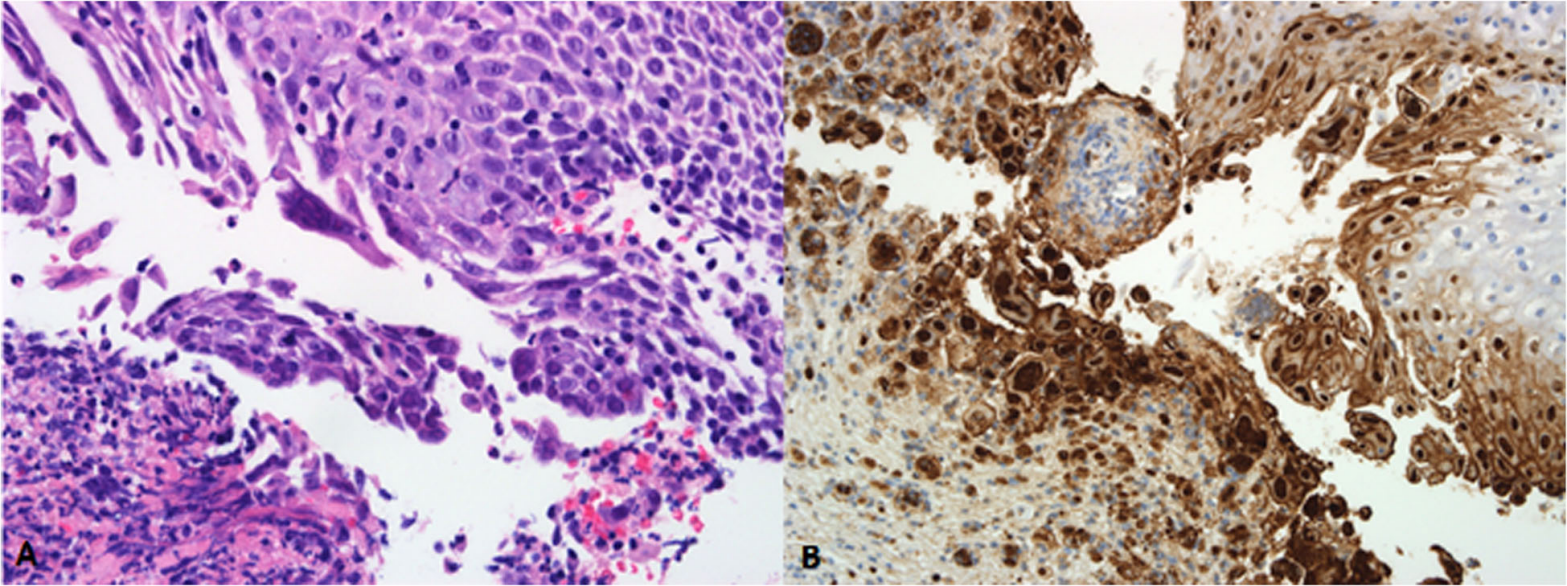
A) The esophageal biopsy shows intraepithelial inflammatory infiltration and multinuclear cells (“coin stack”-like appearance), with cytoplasm of ground-glass appearance and nuclear inclusions suggestive of herpes simplex virus (HSV) infection (hematoxylin-eosin, × 400). B) Immunohistochemistry technique with positive immunostaining demonstrating intranuclear inclusions (anti-HSV-1 antibody, × 200)

Pathological examination can show a wide range of inflammatory disorders such as a neutrophilic inflammation, eosinophilic intranuclear inclusions, or multinucleated giant cells [5]. All our patients had anatomopathological findings compatible with acute infection, both in the hematoxylin-eosin staining and in the immunohistochemistry (Fig. 2), on the other hand, HSV-1 DNA was detected in every patient through polymerase chain reaction [13].

All patients who received acyclovir treatment showed early clinical improvement. In the literature, although the usefulness of acyclovir treatment in immunocompromised is well-defined, its advantage is not yet clear in immunocompetent patients. In these situations, antiviral treatment seemed to reduce the time of disease and prevent complications; however, due to the low incidence of this entity in healthy people, studies have not been carried out to prove the benefit in these individuals [2, 8, 9].

In conclusion, we present four cases of immunocompetent patients with EH who were admitted to our hospital with typical symptomatology and common endoscopical alterations, and in most cases with favorable clinical evolution after antiviral treatment. More studies are necessary in order to understand this pathology in immunocompetent patients. 

## Data Availability

Data sharing is not applicable to this article as no datasets were generated or **analyzed** during the current study.

## Abbreviations

EH: Herpetic esophagitis
HVS: Herpes simplex virus
GERD: Gastroesophageal reflux disease
VZV: Varicela Zoster virus
PCR: Polymerase chain reaction
HIV: Human Immunodeficiency Virus
EoE: Eosinophilic esophagitis
CMV: Cytomegalovirus
ELISA: Enzyme-linked immunosorbent assay
